# Supplementary material for: Mutations in Filamin C Associated with Both Alleles Do Not Affect the Functioning of Mice Cardiac Muscles
Source: Int J Mol Sci. 2025 Feb 7;26(4):1409. doi: 10.3390/ijms26041409 (PMC11855563; doi:10.3390/ijms26041409)
Supplement: Supplementary file 1 [file ijms-26-01409-s001.zip › ijms-3343568-supplementary.pdf]

Supplementary Materials

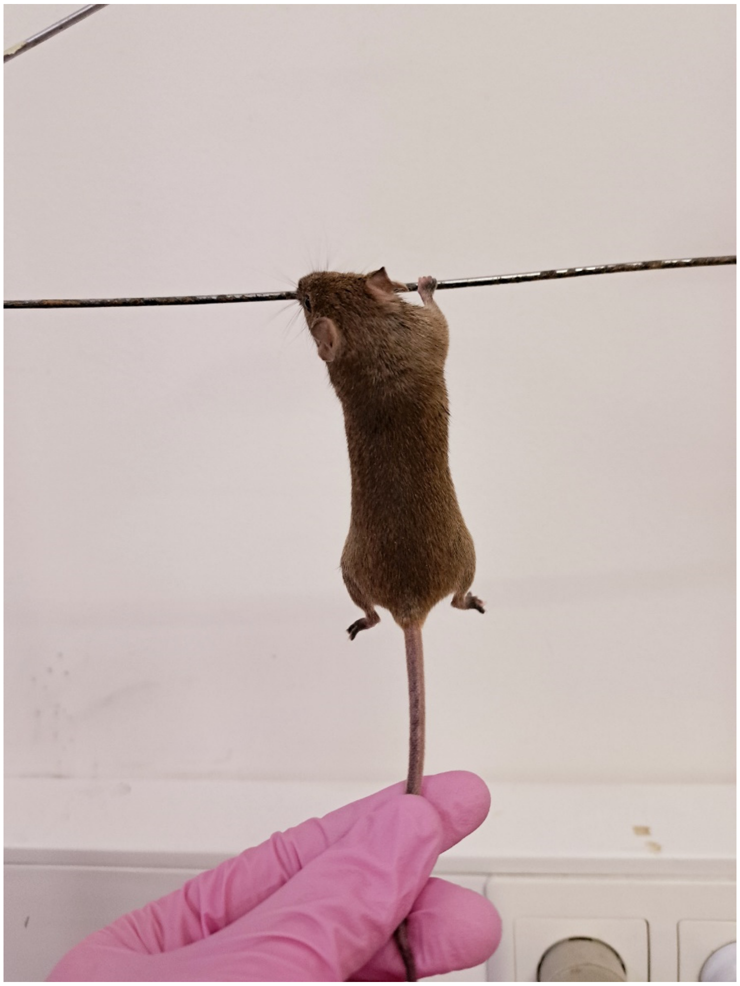

Figure S1. Endurance test performing. Mouse hanging on a wire.

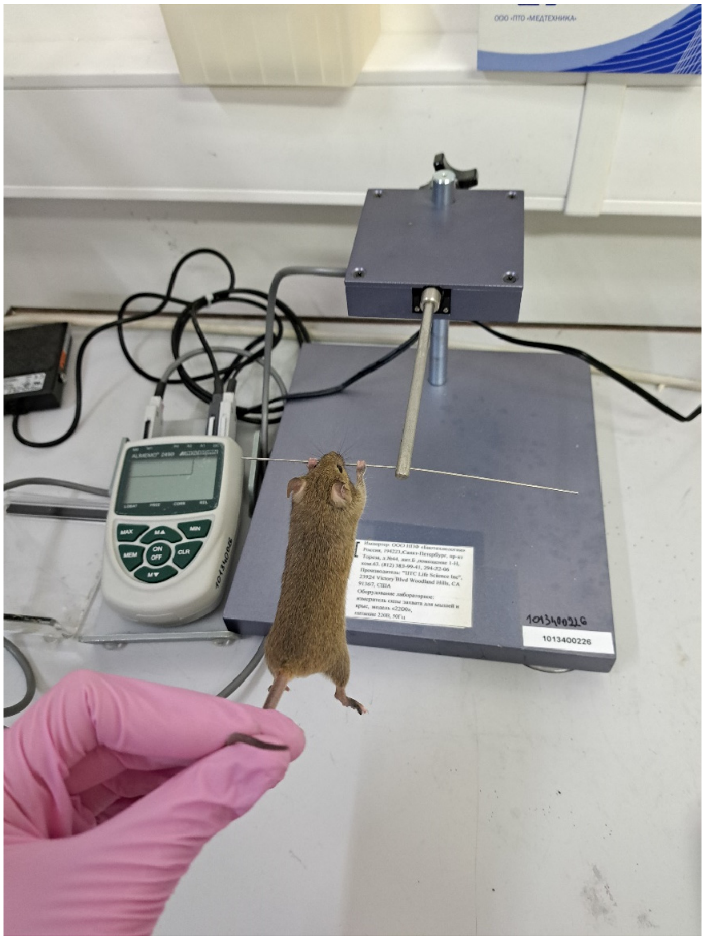

Figure S2. Grip strength test performing. Mouse is grabbing the grid with its forelimbs, while it is slowly pulled backwards.
